# Ecocardiografia Tridimensional Revela o Verdadeiro Inimigo em um Jovem de Sexo Masculino com Infarto do Miocárdio com Supradesnivelamento do Segmento ST e Regurgitação Mitral Grave: “Pseudo-Fenda” Posterior e Prolapso da Valva Mitral

**DOI:** 10.36660/abc.20190485

**Published:** 2021-02-02

**Authors:** Sorina Mihaila, Andreea Elena Velcea, Luigi Paolo Badano, Vinereanu Dragos, Denisa Muraru

**Affiliations:** 1 University of Medicine and Pharmacy Carol Davila Bucharest Emergency University Hospital Bucharest Bucharest Romênia University of Medicine and Pharmacy Carol Davila Bucharest - Emergency University Hospital Bucharest, Bucharest - Romênia; 2 San Luca Hospital Department of cardiac, neural and metabolic sciences Istituto Auxologico Italiano, IRCCS Milão Itália Istituto Auxologico Italiano, IRCCS, Department of cardiac, neural and metabolic sciences, San Luca Hospital, Milão - Itália; 3 University of Milano-Bicocca Department of Medicine and Surgery Milão Itália University of Milano-Bicocca, Department of Medicine and Surgery, Milão - Itália

**Keywords:** Valva Mitral/anormalidades, Infarto do Miocárdio, Ecocardiografia Tridimensional/métodos, Diagnóstico por Imagem, Adulto Jovem

## Introdução

A ecocardiografia tridimensional (E3D) desempenha um papel cada vez mais importante no diagnóstico das valvopatias, na avaliação da morfologia valvar de maneira anatômica e no estabelecimento da reparabilidade da valva, sem ter as limitações da ecocardiografia bidimensional (E2D) convencional.^1^

Nós relatamos o caso de um paciente jovem que apresentou infarto agudo do miocárdio anterior com supradesnivelamento do segmento ST e regurgitação mitral (RM) grave, após intervenção coronária percutânea (ICP) primária bem-sucedida da artéria descendente anterior esquerda, cuja ecocardiografia transesofágica tridimensional (ETE 3D) revelou uma causa inesperada da RM, a saber, patologia complexa da valva mitral (VM) constituindo prolapso dos escalopes P2-3, *flail* de corda e pseudo-fenda do folheto posterior separando o segmento P1 do segmento P2.

## Relado de Caso

Um paciente do sexo masculino, 38 anos de idade, sem fatores de risco cardiovascular conhecidos, apresentou quadro início agudo de dor torácica constritiva. O exame cardíaco revelou ritmo regular, sopro sistólico apical e pressão arterial normal. O eletrocardiograma de 12 derivações em repouso de emergência mostrou elevação do segmento ST nas derivações V_1-6_ e taquicardia ventricular não sustentada recorrente. A angiografia coronária de emergência mostrou oclusão trombótica aguda da artéria descendente anterior esquerda proximal, estenose não crítica da artéria coronária direita e estenose de 90% da artéria circunflexa esquerda. Foi realizada ICP primária com implante de stent na artéria descendente anterior esquerda, com bons resultados procedurais.

**Figura 1:**
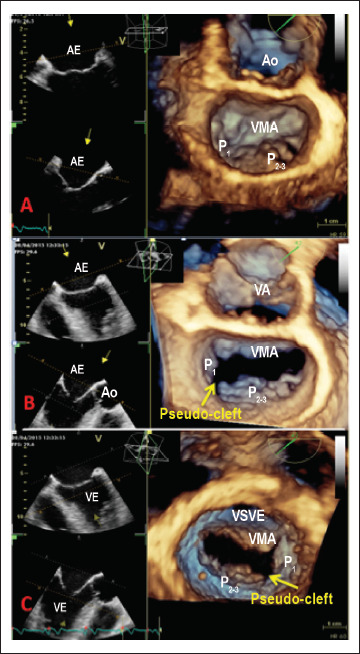
Avaliação morfológica tridimensional da valva mitral (abordagem transesofágica). Painel A) “Visão cirúrgica” da valva mitral fechada, do lado do átrio esquerdo, que mostra o prolapso complexo dos escalopes P_2-3_. O folheto anterior da valva mitral tem morfologia normal. Painel B) Abertura da valva mitral revela que o segmento P_1_ está separado dos segmentos P_2-3_, levantando a suspeita de uma pseudo-fenda. Painel C) Valva mitral visualizada do lado do ventrículo esquerdo. A pseudo-fenda do folheto posterior da valva mitral, entre os segmentos P_1_ e P_2-3_, pode ser identificada. AE: átrio esquerdo, Ao: aorta, RM: regurgitação mitral, VA: valva aórtica, VE: ventrículo esquerdo, VMA: valva mitral anterior, VSVE: via de saída do ventrículo esquerdo.

**Figura 2:**
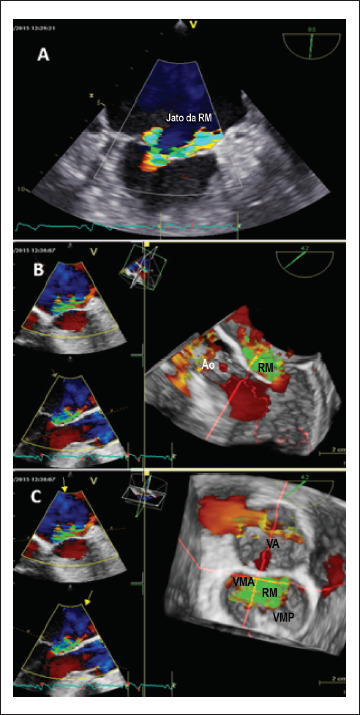
Avaliação bi- e tridimensional em cores da regurgitação mitral (abordagem transesofágica). Painel A) A valva mitral, visualizada a 85 graus, mostra os dois componentes da regurgitação mitral, causada pelo prolapso e pela pseudo-fenda. Painel B) A visão de eixo longo dos escalopes A2/P2 mostra o jato da regurgitação mitral causado pelo prolapso de P2, oposto ao escalope. Painel C) A “visão cirúrgica” da valva mitral mostra a origem ampla do jato da regurgitação mitral, visto do átrio esquerdo, que é direcionado anteriormente (são exibidos apenas os fluxos retrógrados). Ao: aorta; RM: regurgitação mitral, VA: valva aórtica, VMA: valva mitral anterior, VMP: valva mitral posterior.

A E2D transtorácica pós-procedimento mostrou ventrículo esquerdo (VE) não dilatado, anormalidades do movimento da parede septal e discreta disfunção sistólica do VE (fração de ejeção do VE = 50%), bem como dilatação moderada do átrio esquerdo (AE) e RM grave com um jato excêntrico, dirigido anteriormente para o AE (regurgitação holossistólica; área efetiva do orifício regurgitante = 0,4 cm^2^; volume regurgitante = 55 ml/m^2^). Um discreto prolapso do folheto posterior da VM também foi detectado pela ecocardiografia transtorácica bidimensional (ETT 2D). No entanto, nem a anormalidade do movimento da parede septal nem o prolapso da VM, visto por ETT 2D, explicaram completamente a gravidade da RM. Neste contexto, os mecanismos e a gravidade da RM foram mais explorados por meio da ecocardiografia transesofágica, incluindo avaliação por E3D. A avaliação da VM por ETE 3D da “visão cirúrgica” mostrou prolapso dos segmentos P_2-3_ (Figura 1, Painel A), ruptura de corda fixada no folheto posterior da VM e uma indentação profunda na VM posterior (Figura 1, Painel B), levando a um jato regurgitante excêntrico no AE até as veias pulmonares. Com a finalidade de determinar a reparabilidade da VM, foi realizado o exame com avaliação da VM por ETE 3D da visão ventricular (Figura 1, Painel C), onde foi detectado uma pseudo-fenda do folheto posterior, com o escalope P_1_ separados dos segmentos de prolapso P_2-3_. A ETE 2D em cores mostrou um jato “dividido” de RM (Figura 2, Painel A), enquanto a ETE 3D em cores mostrou um jato excêntrico de RM, com origem ampla, direcionado anteriormente (Figura 2, Painéis B e C), explicando melhor o mecanismo da RM.

Foram excluídas potenciais causas adquiridas desses achados morfológicos, tais como trauma prévio da VM, cirurgia ou endocardite infecciosa e o diagnóstico final foi de RM grave devido ao prolapso complexo da VM dos segmentos P_2-3_ e ruptura da corda fixada no folheto posterior da VM, associada a uma pseudo-fenda do folheto posterior entre os segmentos P_1_ e P_2_. O paciente foi encaminhado para parecer cirúrgico, devido ao aumento do AE (mostrando evolução prolongada da RM) e novo aparecimento de sintomas após o evento agudo (dispneia de exercício). Foram realizados com sucesso o reparo da VM, incluindo ressecção do prolapso, sutura da pseudo-fenda da VM e anuloplastia mitral, associados ao enxerto da artéria circunflexa esquerda. No seguimento de três anos, o paciente não apresentou recidiva da RM.

## Discussão

Nosso caso clínico mostra a utilidade da E3D para o diagnóstico e avaliação morfológica de lesões complexas da VM, principalmente quando a etiologia é incerta, bem como o seu papel no planejamento de procedimentos cirúrgicos. A suspeita inicial da etiologia da RM era isquêmica; entretanto, o curto período de isquemia (menos de 2 horas até a revascularização), as pequenas anormalidades no movimento da parede do VE e a boa função sistólica do VE tornaram essa causa improvável. A avaliação detalhada da E2D revelou prolapso leve da VM posterior, que também foi insuficiente para explicar a gravidade da RM. Por outro lado, a ETE 3D revelou o prolapso complexo da VM dos segmentos, a ruptura de corda e a pseudo-fenda do folheto posterior da VM separando o escalope P_1_ do P_2_.

Hipotetiza-se que as fendas são resultado da expressão incompleta de um defeito do coxim endocárdico, na maioria das vezes envolvendo a parte central do folheto anterior da VM.^2^^,^^3^ Fendas verdadeiras que afetam a VM posterior são extremamente raras,^2^ no entanto, as pseudo-fendas são uma classe separada de anomalias morfológicas do folheto posterior da VM. As pseudo-fendas são indentações profundas que compartilham a localização das fendas normais entre os escalopes da VM posterior, mas com mais de 50% da profundidade dos escalopes adjacentes.^4^ Esta anomalia está frequentemente associada à rotação anti-horária dos músculos papilares, músculo papilar acessório ou folheto da VM acessório, e prolapso da válvula mitral.^5^ Nosso paciente apresentou RM como consequência de prolapso complexo da VM com ruptura de corda, associado à pseudo-fenda. As altas pressões diastólicas finais do VE no contexto do evento isquêmico e a disfunção sistólica do VE provavelmente pioraram a gravidade da RM, pois o paciente negava dispneia antes da internação hospitalar. Além disso, permanece a dúvida se a ruptura da corda ocorreu antes ou se estava relacionada ao evento isquêmico.

No entanto, apesar de não ser totalmente responsável pela RM, a presença da pseudo-fenda tem influência adicional na decisão cirúrgica quanto à reparabilidade da VM. Mantovani et al.,^6^ mostraram que 35% dos pacientes com prolapso da VM apresentavam pseudo-fendas, não vistas pela E2D e reveladas apenas pela E3D. A presença de pseudo-fendas não resolvidas em pacientes com prolapso da VM foi associada a pior prognóstico após o reparo da VM e maior recorrência da RM no seguimento. Neste contexto, foi realizado o reparo da VM em nosso paciente, incluindo a sutura da pseudo-fenda da VM.

## Conclusões

A ETE 3D é uma técnica útil e viável para o diagnóstico correto em pacientes com doença complexa da VM, especialmente quando a etiologia é incerta, bem como para a determinação da a reparabilidade da valva. Embora as pseudo-fendas da VM raramente levem à regurgitação, elas estão associadas a piores desfechos pós-operatórios; portanto, precisam ser suturadas durante o reparo da VM.
