# Supplementary material for: Ecocardiografia Tridimensional Revela o Verdadeiro Inimigo em um Jovem de Sexo Masculino com Infarto do Miocárdio com Supradesnivelamento do Segmento ST e Regurgitação Mitral Grave: “Pseudo-Fenda” Posterior e Prolapso da Valva Mitral
Source: Arq Bras Cardiol. 2021 Feb 2;116(2 Suppl 1):36–8. [Article in Portuguese] doi: 10.36660/abc.20190485 (PMC8118632; doi:10.36660/abc.20190485)
Supplement: Translation

# Translation: Three-dimensional Echocardiography Reveals the True Enemy in a Young Male with ST-Elevation Myocardial Infarction and Severe Mitral Regurgitation: Posterior Mitral Valve “Pseudo-Cleft” and Prolapse

**Authors:** Sorina Mihaila, Andreea Elena Velcea, Luigi Paolo Badano, Vinereanu Dragos, Denisa Muraru

**Affiliations:** 1 Bucharest Romania University of Medicine and Pharmacy Carol Davila Bucharest - Emergency University Hospital Bucharest, Bucharest - Romania; 2 Milan Italy Istituto Auxologico Italiano, IRCCS, Department of cardiac, neural and metabolic sciences, San Luca Hospital, Milan - Italy; 3 Milan Italy University of Milano-Bicocca, Department of Medicine and Surgery, Milan - Italy

**Keywords:** Mitral Valve/abnormalities, Myocardial Infarction, Echocardiography, Three Dimensional/methods, Diagnosis Imaging, Young Adult

## Introduction

Three-dimensional echocardiography (3DE) plays an increasingly important role in the diagnosis of valvular heart disease, in the assessment of valvular morphology in an anatomical manner, and in establishing valve repairability, beyond the limitations of conventional two-dimensional echocardiography (2DE).^1^

We report the case of a young patient presenting with acute anterior ST-segment elevation myocardial infarction and severe mitral regurgitation (MR) after successful primary percutaneous coronary intervention (PCI) of the left anterior descending artery, whose three-dimensional transesophageal echocardiography (3D TEE) revealed an unexpected cause of the MR, namely, complex mitral valve (MV) pathology consisting of prolapse of the P_2-3_ scallops, flail chordae, and pseudo-cleft of the posterior leaflet separating the P_1_ from the P_2_ segment.

## Case Report

A 38-year-old male patient, without any known cardiovascular risk factors, presented with acute onset of constrictive thoracic pain. Cardiac examination revealed regular rhythm, apical systolic murmur, and normal blood pressure. Emergency 12-lead resting electrocardiogram showed ST-segment elevation in the V_1-6_ leads and recurrent non-sustained ventricular tachycardia. The emergency coronary angiogram showed acute thrombotic occlusion of the proximal left anterior descending artery, non-critical stenosis of the right coronary artery, and 90% stenosis of the left circumflex artery. Primary PCI with stenting of the left anterior descending artery was performed, with good procedural results.

Post-procedural transthoracic 2DE showed non-dilated left ventricle (LV), septal wall motion abnormalities, and mild LV systolic dysfunction (LV ejection fraction = 50%), as well as moderate dilation of the left atrium (LA) and severe MR with an eccentric jet, directed anteriorly into the LA (holo-systolic regurgitation, effective regurgitant orifice area = 0.4 cm^2^, regurgitant volume = 55 ml/m^2^). A mild prolapse of the posterior MV leaflet was also detected by two-dimensional transthoracic echocardiography (2D TTE). However, neither the septal wall motion abnormality nor the MV prolapse as seen by 2D TTE entirely explained the severity of the MR. In this context, the mechanisms and the severity of the MR were further explored using transesophageal echocardiography, including 3DE assessment. 3D TEE assessment of the MV from the “surgical view” showed prolapse of the P_2-3_ segments (Figure 1, Panel A), a ruptured chordae attached to the posterior MV leaflet, and a deep indentation of the posterior MV (Figure 1, Panel B), leading to an eccentric regurgitant jet into the LA up to the pulmonary veins. To establish MV repairability, the exam was completed with 3DE assessment of the MV from the ventricular view (Figure 1, Panel C), where a pseudo-cleft of the posterior leaflet was detected, with the P_1_ scallop separated from the P_2-3_ prolapsing segments. Color 2D TEE showed a “split” jet of MR (Figure 2, Panel A), while color 3D TEE showed an eccentric MR jet, with wide origin, directed anteriorly (Figure 2, Panel B and Panel C), further explaining the mechanism of the MR.

**Figure 1:** Three-dimensional morphological assessment of the mitral valve (transesophageal approach). Panel A. “Surgical view” of the closed mitral valve, seen from the left atrial side, which shows a complex prolapse of the P2-3 scallops. The anterior mitral valve leaflet shows normal morphology. Panel B. Opening of the mitral valve reveals that the P1 segment is separated from the P2-3 segments, raising the suspicion of a pseudo-cleft. Panel C. The mitral valve visualized from the left ventricular side. The pseudo-cleft of the posterior mitral valve leaflet, between the P1 and the P2-3 segments, can be identified. AMV: anterior mitral valve; Ao: aorta; AoV: aortic valve; LA: left atrium; LV: left ventricle; LVOT: left ventricular outflow tract.

**Figure 2:** Two- and three-dimensional color assessment of the mitral regurgitation (transesophageal approach). Panel A. The mitral valve, visualized at 85 degrees, shows the two components of the mitral regurgitation, caused by the prolapse and the pseudo-cleft. Panel B. Long-axis view throughout the A2/P2 scallops shows the mitral regurgitation jet caused by the prolapse of P2, opposite the scallop. Panel C. The “surgical view” of the mitral valve shows the wide origin of the mitral regurgitation jet, seen from the left atrium, which is directed anteriorily (only retrograde flows are displayed). AMV: anterior mitral valve, Ao: aorta, AoV: aortic valve, MR: mitral regurgitation, PMV: posterior mitral valve.

Potential acquired causes of these morphological findings, such as previous MV trauma, surgery, or infective endocarditis were excluded, and the final diagnosis was severe MR due to complex MV prolapse of the P_2-3_ segments and ruptured chordae attached to the posterior MV leaflet, associated with a pseudo-cleft of the posterior leaflet between the P_1_ and P_2_ segments. The patient was further referred for surgical opinion, due to enlargement of the LA (showing a prolonged evolution of the MR), and new onset of symptoms after the acute event (exercise dyspnea). MV repair, including prolapse resection, suture of the MV pseudo-cleft, and mitral annuloplasty were successfully performed, associated with grafting of the left circumflex artery. At three-year follow-up, the patient showed no recurrence of the MR.

## Discussion

Our clinical case shows the usefulness of 3DE for the diagnosis and morphological assessment of complex MV lesions, especially when the etiology is uncertain, as well as its role in planning surgical procedures. Initial suspicion of the MR etiology was ischemic; however, the short period of ischemia (less than 2 hours until revascularization), minor LV wall motion abnormalities, and good LV systolic function rendered this cause improbable. The careful 2DE assessment revealed mild prolapse of the posterior MV, which was also insufficient to explain the severity of the MR. Conversely, 3D TEE revealed complex MV prolapse of the P_2-3_ segments, a ruptured chordae, and pseudo-cleft of the posterior MV leaflet separating the P_1_ from the P_2_ scallop.

Clefts are hypothesized to be a result of incomplete expression of an endocardial cushion defect, most often involving the middle part of the anterior MV leaflet.^2^^,^^3^ True clefts affecting the posterior MV are extremely rare.^2^ However, pseudo-clefts are a separate class of morphologic anomalies of the posterior MV leaflet. Pseudo-clefts are deep indentations, sharing the localization of normal slits between the scallops of the posterior MV, but with over 50% the depth of adjacent scallops.^4^ This anomaly is frequently associated with counterclockwise rotation of the papillary muscles, accessory papillary muscle or MV leaflet, and MV prolapse.^5^ Our patient presented MR as a consequence of complex MV prolapse with a ruptured chordae, associated with the pseudo-cleft. High LV end-diastolic pressures in the context of the ischemic event and LV systolic dysfunction probably worsened the severity of the MR, as the patient denied any dyspnea prior to hospitalization. Moreover, the question remains whether the rupture of the chordae occurred prior or was related to the ischemic event.

However, even though not entirely responsible for the MR, the presence of the pseudo-cleft has an additional influence on the surgical decision regarding the reparability of the MV. Mantovani et al.^6^ showed that 35% of patients with MV prolapse have pseudo-clefts, not seen by 2DE and revealed only by 3DE. The presence of unsolved pseudo-clefts in patients with MV prolapse was associated with poor prognosis after MV repair and a higher recurrence of the MR at follow-up. In this context, MV repair was performed in our patient, and it included the suture of the MV pseudo-cleft.

## Conclusions

3D TEE is a useful and feasible technique for correct diagnosis in patients with complex MV disease, especially when etiology is uncertain, and for determining valve repairability. Even though MV pseudo-clefts rarely lead to regurgitation, they are associated with worse postoperative outcomes; therefore, they need to be sutured during MV repair.
